# A Rapid Immunoassay for Detection of Shiga Toxin-Producing Escherichia coli Directly from Human Fecal Samples and Its Performance in Detection of Toxin Subtypes

**DOI:** 10.1128/JCM.01785-16

**Published:** 2016-11-23

**Authors:** Jeremy T. Boone, Davina E. Campbell, Amy S. Dandro, Li Chen, Joel F. Herbein

**Affiliations:** TechLab, Inc., Blacksburg, Virginia, USA; Memorial Sloan-Kettering Cancer Center

## Abstract

Fecal samples (*n* = 531) submitted to a regional clinical laboratory during a 6-month period were tested for the presence of Shiga toxin using both a Vero cell cytotoxicity assay and the Shiga Toxin Quik Chek test (STQC), a rapid membrane immunoassay. Testing the samples directly (without culture), 9 positives were identified by the Vero cell assay, all of which were also detected by the STQC. The correlation between the two assays was 100%. Not all of the identified positive samples were detected when fecal broth cultures were tested. By testing broth cultures of characterized isolates representing all described Shiga toxin subtypes, the STQC detected all subtypes. Levels of induction of toxin production by ciprofloxacin differed among the strains tested, with more toxin induction seen in strains harboring Stx2 phages than in those harboring Stx1 phages.

## INTRODUCTION

Since it was first described in the early 1980s, Shiga toxin-producing Escherichia coli (STEC) has been identified as a common cause of foodborne illness both domestically and worldwide, causing an estimated 100,000 illnesses annually in the United States alone (1, 2). In the most severe cases, the disease can progress to life-threatening complications such as hemorrhagic colitis and hemolytic-uremic syndrome (HUS) (3, 4). Early detection of STEC infections is of paramount importance, as the effectiveness of antibiotics that are frequently used to treat other causes of infectious acute diarrhea may be limited, or the use of the antibiotics may even be detrimental, in the treatment of STEC patients (5, 6).

In addition to Shiga toxin production, other virulence factors such as adhesins and intimin are thought to be required for STEC pathogenesis (7, 8). However, as was learned during the 2011 O104:H4 STEC outbreak in Germany, common virulence factors such as intimin, generally present in hypervirulent outbreak strains, need not be present for severe disease to occur (9, 10).

The most common STEC isolate in the United States is O157:H7, frequently detected by stool culture based on its inability to ferment sorbitol within 24 h (11). In recent years, however, the number of non-O157 STEC isolates has increased, resulting in an additional 6 serotypes (O26, O45, O103, O111, O121, and O145) being classified as adulterants by the USDA in 2012 (8, 12, 13). Testing for pathogenic STEC by serotype alone, though, is not an option, as serotype, toxin production, and pathogenic potential are not always linked (14).

The one feature common to all STEC strains is the ability to produce one or both Shiga toxins—Shiga toxin 1 (Stx1) or Shiga toxin 2 (Stx2); therefore, the CDC recommends that all stool samples from patients with acute community-acquired diarrhea be tested for Shiga toxin (15). Stx1 is almost identical to the toxin produced by Shigella dysenteriae, whereas Stx2 is only 56% to 58% homologous. Several subtype variants of each toxin, including three Stx1 variants (Stx1a, Stx1c, and Stx1d) and seven Stx2 variants (Stx2a, Stx2b, Stx2c, Stx2d, Stx2e, Stx2f, and Stx2g), have been identified, and all have been associated with human disease (16–18). Previous investigators have suggested that the available diagnostic assays, both immunological and molecular, fail to detect either all Shiga toxin subtypes or different strains of STEC producing the same subtype and that the presence of the *stx* gene(s) does not always correlate with disease or expression and production of toxin (19–27). Further, the amounts of Shiga toxin expressed can differ greatly between induced and noninduced cultures (28, 29). The Vero cell cytotoxicity neutralization assay is considered the reference standard for detection of Shiga toxin in fecal samples because of its picogram-level analytical sensitivity (30, 31).

In this study, we evaluated the performance of a new rapid immunoassay, the Shiga Toxin Quik Chek test (STQC), for the detection of Shiga toxin-producing Escherichia coli in human fecal specimens and compared the results to those of a Vero cell cytotoxicity assay using both clinical fecal samples and cultures of isolates representing all described Shiga toxin subtypes. The STQC was able to detect all described Stx1 and Stx2 (Stx1/2) subtypes and correlated 100% with the Vero cell assay in the clinical study.

(Part of this research was presented as a poster at the 54th Interscience Conference on Antimicrobial Agents and Chemotherapy, 5 to 9 September 2014, Washington, DC [32].)

## MATERIALS AND METHODS

### Subtype study.

The STEC isolates used for the subtype study are listed (see Table 2). For each strain, an isolated colony from a blood agar plate (Hardy Diagnostics, Santa Maria, CA) was used to inoculate 5 ml tryptic soy broth (TSB) (Fluka, St. Louis, MO). The TSB culture was incubated at 37°C with 220 rpm shaking, and when it reached mid-log phase (determined by absorbance at 600 nm), 0.4 ml was used to inoculate 8 ml Gram-negative (GN) broth (Becton Dickinson, Sparks, MD). Following overnight (16 to 20 h) stationary incubation at 37°C, the GN broth culture was tested using the STQC (TechLab, Blacksburg, VA) per the package insert procedure. Toxin production was confirmed by Vero cell cytotoxicity assay (33), and positive samples were neutralized with specific rabbit antisera against Stx1 and Stx2 (TechLab, Inc. Blacksburg, VA) to confirm that the cytotoxicity was due to Shiga toxin. The in-house Vero cell assay detected Stx1 and Stx2 at levels of 60 pg/ml and 30 pg/ml, respectively. The Shiga toxin subtypes were confirmed by real-time PCR using a modification of the procedure described by Scheutz et al. (18). Table 1 lists the primers and amplification conditions utilized for the subtyping PCR studies.

**TABLE 1:** Primer sequences and amplification conditions used for subtyping^*a*^

Target	Primer	Primer sequence (5′–3′)	Ampicon size (bp)	Amplification conditions^*b*^
*stx*_1a_	Fwd	CGCGAGTTGCCAGAATGGCATCTG	155	95°C for 10 min; 30 cycles of 95°C for 20 s, 65°C for 20 s, and 72°C for 30 s
	Rev	CATTTTACCCCCTCAACTGC		
*stx*_1c_	Fwd	CCTTTCCTGGTACAACTGCGGTT	252	95°C for 10 min; 30 cycles of 95°C for 20 s and 66°C for 40 s
	Rev	CAAGTGTTGTACGAAATCCCCTCTGA		
*stx*_1d_	Fwd	CAGTTAATGCGATTGCTAAGGAGTTTACC	203	95°C for 10 min; 35 cycles of 95°C for 20 s, 64°C for 20 s, and 72°C for 30 s
	Rev	CTCTTCCTCTGGTTCTAACCCCATGATA		
*stx*_2a_	Fwd	GCGATACTGRGBACTGTGGCC	347	95°C for 10 min; 35 cycles of 95°C for 20 s and 65°C for 60 s
	Rev	GCCACCTTCACTGTGAATGTG		
*stx*_2b_	Fwd	AAATATGAAGAAGATATTTGTAGCGGC	251	95°C for 15 min; 35 cycles of 94°C for 50 s, 64°C for 40 s, and 72°C for 60 s
	Rev	CAGCAAATCCTGAACCTGACG		
*stx*_2c_	Fwd	GAAAGTCACAGTTTTTATATACAACGGGTA	177	95°C for 10 min; 30 cycles of 95°C for 20 s and 67.8°C for 60 s
	Rev	CCGGCCACYTTTACTGTGAATGTA		
*stx*_2d_	Fwd	AAARTCACAGTCTTTATATACAACGGGTG	179	95°C for 10 min; 30 cycles of 95°C for 20 s and 65°C for 60 s
	Rev	TTYCCGGCCACTTTTACTGTG		
*stx*_2e_	Fwd	CGGAGTATCGGGGAGAGGC	266	95°C for 10 min; 30 cycles of 95°C for 20 s, 54°C for 20 s, and 72°C for 30 s
	Rev	TCATTCACCAGTTGTATATAAAGG		
*stx*_2f_	Fwd	TGACGGCTCAGGATGTTGAC	136	95°C for 10 min; 30 cycles of 95°C for 20 s and 60°C for 20 s
	Rev	GCAACACTTCCGAGAATCGC		
*stx*_2g_	Fwd	CACCGGGTAGTTATATTTCTGTGGATATC	573	95°C for 15 min; 35 cycles of 94°C for 50 s, 64°C for 40 s, and 72°C for 60 s
	Rev	GATGGCAATTCAGAATAACCGCT		

a Subtyping was performed using a modified version of the PCR method described in reference 18.

b A melting curve following amplification was used to verify the amplicon.

### Clinical study.

Anonymous unlinked excess fecal samples that had been submitted to a regional clinical laboratory for routine testing during a 6-month period from August 2013 through February 2014 were used for this study. On the day that the samples were received, the Vero cell cytotoxicity neutralization assay was started and the specimens were tested with the STQC per the package insert procedure for direct testing of fecal samples. Because the toxins produced by Clostridium difficile also cause rounding of Vero cells, cell rounding caused by C. difficile toxin was ruled out by neutralization with C. difficile-specific goat antisera (TechLab, Inc. Blacksburg, VA). To obtain STEC isolates for further characterization, as soon as a positive sample was identified by either the STQC or Vero cell assay, GN broth, MacConkey broth (Remel, Lenexa, KS), and sorbitol-MacConkey agar (SMAC) plate (Becton Dickinson) cultures were started. Following overnight (16 to 20 h) incubation at 37°C, broth cultures were tested for toxin by Vero cell assay and broth and SMAC plate cultures were tested with the STQC following the package insert procedure for culture testing. E. coli O157-positive samples were identified using a combination of identification of clear colonies on SMAC plate cultures and an in-house O157 immunoassay using specific monoclonal antibodies.

### Limit of detection (LOD) study.

The STEC isolates used for the LOD study are listed (see Table 5). For each strain, an isolated colony from a blood agar plate was used to inoculate 5 ml TSB broth. The TSB culture was incubated at 37°C with 220 rpm shaking, and when the culture reached mid-log phase (determined by absorbance at 600 nm), 0.4 ml was used to inoculate two tubes, each containing 8 ml GN broth. One of the GN broth tubes contained 15 ng/ml ciprofloxacin (Sigma Chemical, St. Louis, MO), and the other served as a noninduced control (34). Following overnight (16 to 20 h) stationary incubation at 37°C, serial 10-fold dilutions of the cultures were prepared in GN broth and plated on blood agar plates for determination of CFU counts per milliliter. Subsequently, the 10-fold culture dilutions were tested with the STQC per the package insert procedure. Serial 2-fold dilutions in GN broth of the last positive 10-fold dilution were prepared and tested on the STQC to determine the LOD. Toxin production was confirmed by Vero cell cytotoxicity neutralization assay using undiluted culture filtrate.

## RESULTS

### Subtype study.

A panel of 24 STEC strains representing all described Shiga toxin subtypes was utilized for the subtype study (Table 2). With the exception of Stx2c, at least 2 strains representing each unique subtype were tested. Strain 031 produces both Stx2b and Stx2c, and whether either one or both toxins were expressed was not determined. The Vero cell assay, subtyping PCR, and STQC results agreed for all Stx1 subtypes. Some differences were seen, however, among the Stx2 subtype strains. Toxins produced by Stx2a, Stx2c, Stx2d, Stx2e, Stx2f, and Stx2g strains were detected by both the Vero cell assay and the STQC. Of the 4 Stx2b strains, toxin was detected by the Vero cell assay and STQC in only 2, suggesting that either the Stx2b gene was not expressed in strains DG131/3 and EH250 or the amount of toxin produced under the growth conditions used for this study was below the detection limit of the assays.

**TABLE 2:** Detection of Shiga toxin subtypes^*a*^

Strain	Source	Serotype	Subtype	Vero cell assay	STQC result	
					Stx1	Stx2
DEC10B	MSU	O26:H11	Stx1a	Stx1	+	−
TW08101	MSU	O103:H2	Stx1a	Stx1	+	−
DEC8E	MSU	O111:H8	Stx1a	Stx1	+	−
DG131/3	SSI	O174:H8	Stx1c/2b	Stx1	+	−
C296-09	SSI	O22:H8	Stx1c/2b	Stx1/2	+	+
MHI813	SSI	O8:HR	Stx1d	Stx1	+	−
C695-03	SSI	O154:H31	Stx1d	Stx1	+	−
G5506	MSU	O104:H21	Stx2a	Stx2	−	+
TW08023	MSU	O121:H19	Stx2a	Stx2	−	+
86-24	MSU	O157:H7	Stx2a	Stx2	−	+
EH250	SSI	O118:H2	Stx2b	−	−	−
C43-03	SSI	O174:H8	Stx2b	Stx2	−	+
031	SSI	O174:H21	Stx2b/2c	Stx2	−	+
C193-09	SSI	O157:H7	Stx2c	Stx2	−	+
B2F1	MSU	O91:H21	Stx2d	Stx2	−	+
C404-09	SSI	O?:H?	Stx2d	Stx2	−	+
S1191	USU	O139:H1	Stx2e	Stx2	−	+
C289-03	SSI	O23:H12	Stx2e	Stx2	−	+
T4/97	SSI	O128:H2	Stx2f	Stx2	−	+
C548-06	SSI	O145:H34	Stx2f	Stx2	−	+
7v	SSI	O2:H25	Stx2g	Stx2	−	+
C136-03	SSI	O36:H14	Stx2g	Stx2	−	+
EDL-933	MSU	O157:H7	Stx1a/2a	Stx1/2	+	+
94C	SSI	O48:H21	Stx1a/2a	Stx1/2	+	+

a Overnight GN broth cultures were tested for the presence of Shiga toxin with the STQC following the package insert procedure. Shiga toxin production was confirmed by Vero cell assay. MSU, Thomas S. Whittam STEC Center, Michigan State University, East Lansing, MI, USA; SSI, Statens Serum Institut, Copenhagen, Denmark; USU, Allison O'Brien, Uniformed Services University of the Health Sciences, Bethesda, MD, USA.

In an additional set of experiments (data not shown), induction of strain EH250 with 0.5 μg/ml mitomycin C (Santa Cruz Biotechnology, Santa Cruz, CA) stimulated production of Stx2b, which was detected by both the Vero cell assay and the STQC. However, neither mitomycin C nor ciprofloxacin induced Stx2b production in strain DG131/3. Interestingly, a MacConkey broth culture of strain DG131/3 was positive for both Stx1 and Stx2 by the STQC, but a GN broth culture that had been inoculated with the same starter culture used for the MacConkey broth culture was positive only for Stx1.

### Clinical study.

During the 6-month study period, a total of 531 samples were received. Results are summarized in Table 3. The Vero cell cytotoxicity neutralization assay identified 9 positive samples, including 1 sample that was positive for both Stx1 and Stx2, representing a prevalence rate of 1.7%. Direct fecal testing of the samples with the STQC identified the same 9 specimens as Shiga toxin positive, resulting in a correlation of 100% with the Vero cell assay results. Table 4 summarizes additional testing performed with the samples identified as positive by direct fecal testing. Culturing was performed on identified positive stool samples so that pure isolates could be obtained and characterized. Surprisingly, the culture results did not always agree with the direct fecal testing results obtained with the Vero cell assay and the STQC. Sample S1 was detected by direct fecal testing but not by broth or SMAC plate culture, whereas sample S4 was detected by direct fecal and GN broth testing but not by MacConkey broth (no growth) or SMAC plate culture. Additionally, sample S3 was positive only for Stx2 when the fecal sample was tested directly but was positive for Stx1/2 when the fecal sample was cultured. Further subculturing and analysis yielded two distinct isolates from sample S3—an O157/Stx1a/Stx2a strain and a non-O157/Stx1c strain. Except for the Stx1c isolate from sample S3, all of the isolates were of the common Stx1a subtype or the Stx2a subtype or both.

**TABLE 3:** Summary of direct fecal testing results

Parameter	Value			
	Shiga toxin 1 (*n* = 531)		Shiga toxin 2 (*n* = 531)	
	Vero^+^	Vero^−^	Vero^+^	Vero^−^
No. of STQC^+^ samples	7^*a*^	0	3^*a*^	0
No. of STQC^−^ samples	0	524	0	528
Sensitivity (%)	100.0		100.0	
Specificity (%)	100.0		100.0	
PPV^*b*^ (%)	100.0		100.0	
NPV^*c*^ (%)	100.0		100.0	
Correlation (%)	100.0		100.0	

a Nine positive samples were identified, one of which was positive for both Stx1 and Stx2, resulting in 10 positive results.

b PPV, positive predictive value.

c NPV, negative predictive value.

**TABLE 4:** Comparison of direct fecal and broth culture test results and further characterization of isolates from identified positive specimens

Sample	Direct fecal test result				Broth culture result^*a*^				SMAC plate result			Subtype
	STQC		Vero assay		STQC		Vero assay		STQC			
	Stx1	Stx2	Stx1	Stx2	Stx1	Stx2	Stx1	Stx2	Stx1	Stx2	O157	
S1	+	−	+	−	−	−	tnp	tnp	−	−	−	Stx1a
S2	+	−	+	−	+	−	+	−	+	−	−	Stx1a
S3	−	+	−	+	+	+	+	+	+	+	+/−^*b*^	Stx1a/1c/2a
S4	+	+	+	+	+	+	+	+	−	−	+	Stx1a/2a
S5	+	−	+	−	+	−	+	−	+	−	−	Stx1a
S6	+	−	+	−	+	−	+	−	+	−	−	Stx1a
S7	+	−	+	−	+	−	+	−	+	−	−	Stx1a
S8	−	+	−	+	−	+	−	+	−	+	+	Stx2a
S9	+	−	+	−	+	−	+	−	+	−	−	Stx1a

a Broth culture results shown are for GN specimens and agreed with the MacConkey results for all specimens except sample S4, which did not grow in MacConkey broth. tnp, test not performed.

b Two STEC isolates were recovered: one was O157 positive, the other O157 negative.

### Limit of detection (LOD) study.

Because all of the positive samples identified during our clinical study were subtypes Stx1a, Stx1c, and/or Stx2a, characterized clinical isolates representing these 3 subtypes were chosen for a LOD study comparing noninduced to ciprofloxacin-induced GN broth cultures. Ciprofloxacin was chosen as the inducing agent for the LOD studies because it induces Shiga toxin production both *in vivo* and *in vitro* and as such is contraindicated for use in treatment of STEC infections (6, 34–36). Table 5 summarizes the results of the LOD study. Only one of the three Stx1a-only strains, DEC8E, was sensitive to ciprofloxacin, showing a 1.4 log reduction in CFU counts per milliliter. The CFU counts per milliliter for the other two Stx1a strains were not appreciably affected by the addition of the antibiotic. The highest dilution detectable ranged from 1/40 to 1/100, depending on the strain or the presence of ciprofloxacin.

**TABLE 5:** Limit of detection of the STQC—comparison of ciprofloxacin induced and noninduced STEC broth cultures

Strain	Serotype	Subtype	Induced	Vero cell assay	Culture CFU/ml^*a*^	STQC (CFU/test)^*b*^
DEC10B	O26:H11	Stx1a	No	Stx1	1.8 × 10^8^	1.2 × 10^5^
			Yes	Stx1	2.3 × 10^8^	1.4 × 10^5^
TW08101	O103:H2	Stx1a	No	Stx1	3.3 × 10^8^	2.1 × 10^5^
			Yes	Stx1	5.6 × 10^8^	8.8 × 10^5^
DEC8E	O111:H8	Stx1a	No	Stx1	9.0 × 10^8^	1.4 × 10^6^
			Yes	Stx1	3.5 × 10^7^	2.2 × 10^4^
DG131/3	O174:H8	Stx1c/2b	No	Stx1	2.2 × 10^8^	6.3 × 10^5^
			Yes	Stx1	0^*c*^	+
C296-09	O22:H8	Stx1c/2b	No	Stx1/2	1.2 × 10^8^	9.4 × 10^5^ Stx1, 7.5 × 10^5^ Stx2
			Yes	Stx1/2	2.4 × 10^8^	7.5 × 10^5^ Stx1, 7.5 × 10^3^ Stx2
G5506	O104:H21	Stx2a	No	Stx2	3.7 × 10^8^	2.3 × 10^5^
			Yes	Stx2	8.3 × 10^8^	2.6 × 10^4^
TW08023	O121:H19	Stx2a	No	Stx2	6.0 × 10^8^	1.9 × 10^5^
			Yes	Stx2	1.7 × 10^8^	5.3 × 10^3^
86-24	O157:H7	Stx2a	No	Stx2	8.7 × 10^8^	5.4 × 10^5^
			Yes	Stx2	2.1 × 10^8^	1.3 × 10^3^
EDL-933	O157:H7	Stx1a/2a	No	Stx1/2	6.7 × 10^8^	1.0 × 10^6^ Stx1, 5.2 × 10^5^ Stx2
			Yes	Stx1/2	3.2 × 10^5^	20 Stx1, 0.5 Stx2
94C	O48:H21	Stx1a/2a	No	Stx1/2	2.3 × 10^8^	3.6 × 10^5^ Stx1, 7.2 × 10^4^ Stx2
			Yes	Stx1/2	0^*c*^	+ (Stx1, Stx2)

a Data representing CFU counts per milliliter are from the undiluted overnight GN broth culture.

b Data representing CFU counts per test correspond to the LOD determined after factoring in both the broth dilution and the additional dilution that occurs in performing the STQC testing procedure. For cases in which the CFU counts per milliliter could not be determined due to the lack of viable cells, a plus sign (+) indicates that toxin was detected by the assay.

c Although ciprofloxacin was toxic to these strains, toxin was still produced and detected by both the Vero cell assay and the STQC.

The two Stx1c/2b strains responded quite differently to the antibiotic. Ciprofloxacin was toxic to strain DG131/3, as the induced culture was not cloudy following overnight incubation (no growth) and no viable cells remained. Strain C296-09, on the other hand, was unaffected by the antibiotic. Strain C296-09, which was not sensitive to ciprofloxacin, showed no appreciable difference in the LOD between the induced and noninduced cultures for Stx1c, but Stx2b was detectable at a 200-fold-higher dilution in the induced culture, resulting in a LOD for Stx2b that was 2 logs lower. A CFU/test LOD could not be determined for the induced DG131/3 strain, as the cells did not grow; however, the lowest dilution at which toxin was detectable was 1 log higher in the induced culture, suggesting that ciprofloxacin did not increase toxin production.

Toxin production was stimulated by the addition of ciprofloxacin to all 3 strains harboring only Stx2a phage. The antibiotic was not toxic to any of the Stx2a strains. Toxin was detected in induced cultures of G5506 and TW08023 at dilutions that were 20-fold and 10-fold higher, respectively, than those seen with their noninduced counterparts. Strain 86-24, an O157:H7 serotype strain, showed the largest increase in toxin production. The induced culture could be detected at a 1/10,000 dilution, representing a 2 log increase compared to the results determined for the noninduced control.

Both the Stx1a and Stx2a strains were sensitive (growth impaired) to ciprofloxacin: a 3.3 log drop in CFU/ml was seen in the induced EDL-933 culture. Stx1a was detectable at a 25-fold-higher dilution and Stx2a at a 500-fold-higher dilution, resulting in LODs for Stx1a and Stx2a that were 4.7 log and 6 log lower, respectively, in the induced EDL-933 culture. No viable cells remained in the induced 94C culture, and there was no visible growth. Because the lowest dilutions at which toxin was detectable were similar for Stx1a and Stx2a in both the induced and noninduced cultures, ciprofloxacin did not induce toxin production in this strain.

## DISCUSSION

Willford et al. and Feng et al. reported in 2009 and 2011, respectively, that the Shiga toxin immunoassays commercially available at the time failed to detect all known Shiga toxin subtypes (19, 27). Furthermore, as *stx*_2f_ and *stx*_2a_ are only 60% similar at the nucleic acid level, *stx*_2f_ is not detected by many PCR methods, including those used by Feng et al. (21). Because the STQC was not available at the time that the Feng and Willford papers were published, our group proposed a similar study to evaluate the performance of the STQC with all described Stx subtypes.

STEC strains that produce only Stx1 are generally associated with mild disease, whereas strains that produce Stx2 are associated with more-severe disease (30, 37). While it has been reported that subtypes Stx2a and Stx2c are more frequently associated with HUS than other Stx2 subtypes, Stx2b, Stx2d, and Stx2e strains have also been isolated from the stools of patients with HUS; therefore, it is important for diagnostic assays to be able to identify these Shiga toxin subtypes as well (10, 24, 38, 39). A review of several clinical studies in which the STEC isolates were characterized revealed that the vast majority (95%) were Stx2a, Stx2c, Stx1a, or Stx2d or some combination thereof. The remainder were Stx1c (3%), Stx2e (2%), and Stx2f (0.1%) (10, 24, 38, 40). Stx2f is usually associated with avian species, although it has occasionally been isolated from humans with mild gastroenteritis (41–43). STEC isolates harboring Stx2g phage, first described as a bovine isolate with high homology to Stx2a and Stx2c, have also been collected from human fecal samples, including isolates containing the intimin gene (*eae*); therefore, Stx2g STEC is also a potential human pathogen capable of disease (38, 44, 45).

The STQC detected all described Shiga toxin subtypes. One of the two Stx2b subtypes (EH250) did not produce detectable toxin in broth, as it was negative by both the Vero cell assay and STQC. Using different culture conditions, including mitomycin C induction, however, Beutin et al. were able to detect with their Vero cell assay toxin produced by strain EH250, suggesting, as we have also observed, that toxin production varies depending upon broth type, inducing agent, and culture conditions (20). Recently, two independent groups (25, 26) reported that the STQC was unable to detect all described Shiga toxin subtypes. In both of those studies, however, the immunoassay results were compared to those of a real-time PCR assay and toxin production was not verified by Vero cell assay.

During the clinical study, the STQC detected all 9 of the positive samples identified by the Vero cell assay. The Vero cell cytotoxicity neutralization assay is considered the gold standard for the detection of Shiga toxin in fecal filtrate, and while extremely sensitive (30 to 60 pg/ml LOD), it is laborious and results are not available for 48 to 72 h. Of the 9 identified positive specimens, only 8 were detected by GN broth culture testing, 7 by MacConkey broth culture testing, and 7 by testing colonies from a SMAC plate culture. Samples that test positive by the direct fecal method but negative when cultured could be explained by a lack of viable STEC cells in the fecal specimen, a low number of STEC cells that are outcompeted by other fecal organisms when cultured, or the presence of inhibitors, such as antibiotics, in the fecal sample. No false-positive results were seen with the STQC compared to the Vero cell assay, resulting in a specificity of 100%. High specificity is important for assays targeting diseases with low prevalence rates, such as those caused by STEC; a specificity of <99% has a detrimental effect on the positive predictive value. Our values of 100% sensitivity and 100% specificity with direct testing of fecal samples are higher than those reported recently by other groups for the STQC (25, 26). However, the two previous studies used a real-time PCR assay as the reference method instead of a Vero cell cytotoxicity assay, and expression of Shiga toxin genes was not confirmed. In two separate comparisons to PCR, Chui et al. demonstrated enhanced performance with the STQC in comparison to another commercial rapid immunoassay specific for Shiga toxin detection in fecal broth cultures—the authors reported sensitivity/specificity of 85%/100% for the STQC versus 35%/99% for the other rapid immunoassay (25, 46). Previous studies by other groups comparing a commercially available microplate enzyme-linked immunosorbent assay (ELISA) to Vero cell cytotoxicity assays reported sensitivities of 40% to 83.9% and specificities of 76.9% to 99.8% for direct testing of stool specimens (47, 48).

Two STEC isolates (an O157/Stx1a/Stx2a strain and a non-O157/Stx1c strain) were obtained from sample S3. While those results were unusual, others have reported multiple STEC isolates from the same patient sample (10, 49). Interestingly, this specimen was Stx2 positive only when tested directly, even though both Stx1 and Stx2 were detected when the sample was cultured. It is possible that Stx1 production was not stimulated *in vivo* whereas Stx2 production was stimulated (50). In addition to antibiotics, other agents present in the gut, such as bacteriocins, and neutrophil products, such as peroxides, can induce toxin production (51, 52). Because toxin-producing strains have frequently been isolated from asymptomatic carriers, *in vivo* toxin production and *in vitro* toxin production may not always correlate (40, 53, 54).

It has been well documented that *in vitro* Shiga toxin production varies depending on the culture conditions and isolate (28, 36, 55, 56). Our results indicate that Stx2 phages in the strains studied are generally more susceptible to induction with ciprofloxacin than are Stx1 phages. With the exception of EDL-933, none of the Stx1 phages were induced by ciprofloxacin. In contrast, all of the Stx2 strains that expressed toxin (with the exception of 94C, which did not grow) were induced by the addition of ciprofloxacin. Phage susceptibility to ciprofloxacin induction seems to be attributable to some extent to the phage as well as to the bacterial host, as evidenced by strain C296-09, which hosts both Stx1c and Stx2b phages. In this strain, Stx1 production was unaffected by the antibiotic, whereas Stx2 production was increased 200-fold. Since strains DEC10B, TW08101, DEC8E, and EDL-933 all host Stx1a phages, it is likely that the Stx1a phage in EDL-933 either is a different phage variant or is integrated at a different chromosomal location, thereby explaining the differences in induction (57).

Others have found that E. coli DH5α and Shigella sonnei lysogens infected with Stx2 phages do not always express toxin in the same manner as the STEC strain from which the phages were originally isolated, suggesting that both phage and host factors, which could include additional bacteriophages, can influence Shiga toxin expression and inducibility (58, 59). Additionally, other research groups have shown that different E. coli serotypes respond differently to ciprofloxacin induction of Stx phages, with O157 isolates having higher inducibility, which agrees with our findings (28). It is also possible that Stx phages lacking the *rusA* Holliday junction resolvase sequence, such as the EDL-933 Stx1a phage, may be more amenable to induction by ciprofloxacin in hosts resistant to the antibiotic.

The broth culture LOD studies described here were not performed in the presence of fecal extract. Both we and others have seen that competing microorganisms, antibiotics, and other agents that might be present in a fecal sample can either increase or decrease Shiga toxin production in broth culture (28, 29, 51, 52, 60). Therefore, the LODs determined here may or may not be reflective of an LOD determined in the presence of any particular fecal extract, and testing in the presence of those fecal extracts may not be reflective of actual *in vivo* conditions. In our study, we chose to focus on controlled conditions in order to eliminate inconsistencies.

Whereas it is difficult to determine the amount of STEC bacteria present in a fecal sample due to the high number of other bacteria present, serotype O157 can be enumerated on selective media such as sorbitol-MacConkey agar. Reported levels of O157 STEC in human fecal specimens range from 1.8 × 10^7^ CFU/g to 2 × 10^9^ CFU/g (61). Two O157:H7 isolates (86-24 and EDL-933) were included in our *in vitro* LOD studies. Extrapolating from the CFU/test LOD values reported in Table 5 to approximate the CFU count per gram that would be present in a fecal sample, the STQC can detect 4.8 × 10^6^ CFU/g to 6.6 × 10^7^ CFU/g based on the noninduced LODs and 3.3 × 10^1^ CFU/g to 1.3 × 10^3^ CFU/g based on the ciprofloxacin-induced LODs. Whereas *in vitro* and *in vivo* levels of toxin production may differ, the CFU counts per gram extrapolated from the LOD studies suggest that the STQC can detect clinically relevant levels of STEC.

In summary, infections caused by STEC are complex scenarios involving mobile phages, with Shiga toxin expression influenced by a combination of phage, bacterial, and human host factors. The STQC is capable of detecting all described Shiga toxin subtypes. In a clinical study in which fecal samples were tested directly (without culture), performance of the STQC was comparable to that of a Vero cell cytotoxicity assay, identifying positive samples that were not detected when cultured. Because both phage and bacterial host factors as well as culture conditions influence Shiga toxin expression levels *in vitro*, fecal broth cultures may not always accurately reflect *in vivo* Shiga toxin production in patients infected with STEC.
